# Genetic polymorphisms of superoxide dismutase-1 A251G and catalase C-262T with the risk of colorectal cancer

**Published:** 2017-06

**Authors:** Iman Jamhiri, Iraj Saadat, Shahpour Omidvari

**Affiliations:** 1 Department of Biology, College of Sciences, Shiraz University, Shiraz, Iran; 2 Department of Chemotherapy, Shiraz University of Medical Sciences, Shiraz, Iran

**Keywords:** SOD1, Catalase, Colorectal cancer, Genetic polymorphism

## Abstract

Oxidative stress is significant in numerous types of disease including cancer. To protect cells and organs against reactive oxygen species (ROS), the body has evolved an antioxidant protection system that involved in the detoxification of ROS. Single nucleotide polymorphisms (SNP) of anti-oxidative enzymes may dramatically change the activity of the encoded proteins; therefore, certain alleles can be established as risk factors for some kind of multi-factorial diseases including cancer. In present study we investigate the possible association between polymorphisms of superoxide dismutase 1 (*SOD1*, OMIM: 147450) and catalase (*CAT*, OMIM: 115500) genes and the risk of colorectal cancer (CRC). The study included 204 colorectal cancer patients and 239 healthy control group matched for gender and age. Genotyping of *SOD1 *A251G and *CAT *C-262T were done by polymerase chain reaction and restriction fragment length polymorphism (PCR-RFLP) method. There was no significant association between *CAT *C-262T polymorphism and susceptibility to CRC (P>0.05). The carries of the G allele of *SOD1 *significantly showed higher prevalence in CRC patients compared with the control group (OR=1.84, 95% CI=1.13-2.98, P=0.013). We assessed the effect of combination of genotypes of the study polymorphisms on the risk of CRC. We found that the combination of AG+GG (*SOD1*) and CC (*CAT*) increases the risk of developing CRC (OR=2.38, 95% CI=1.25-4.52, P=0.008).

## INTRODUCTION

Colorectal cancer (CRC) is one of the most serious public health problems. There are nearly one million cases of CRC diagnosed worldwide each year. CRC is the third and the fifth most common cancer in women and men respectively in Iran [1]. Etiological studies have attributed that CRC risk increased by several environmental factors such as red meat consumption, cigarette smoking and exposure to carcinogenic aromatic [2-5]. Interest in potential genetic variants in antioxidant pathways and disease progression has been increased [6]. The enzymes that are usually considered to be the frontline defense against reactive oxygen species (ROS) are through superoxide dismutase (SOD) and catalase (CAT). SODs are a family of enzymes responsible for converting of superoxide into hydrogen peroxide. The *SOD1 *gene (OMIM: 147450) is located on chromosome 21p22.11 and contains 5 exons and 4 introns (7). Catalase is an enzyme that causes the decomposition of hydrogen peroxide into water and oxygen, thus preventing cells from high levels of ROS [8]. The *CAT *gene (OMIM: 115500) is placed on chromosome 11p13.31 and contains 13 exons.

Genetic variations in the antioxidant genes coding for the *SOD *and *CAT *may lead to decreased or impaired regulation of their enzymatic activity and alter ROS detoxification. Therefore, genetic variations among enzymes that protect the cell against ROS may modulate disease risk [9]. Among the three isoforms of *SOD *(*SOD*1, CuZn- *SOD*; *SOD*2, Mn-*SOD*; and *SOD*3, EC-*SOD*), *SOD*1 plays an important role as an intracellular cytoplasmic antioxidant enzyme and 251A/G sequence variant in intron 3 has been studied most frequently [7]. Also polymorphisms of *CAT *are associated with many cancers [10]. The C-262T polymorphism is a common variant in the promoter region of the *CAT*. The purpose of the present study was to evaluate the association of *SOD1 *A251G and *CAT *C-262T genetic polymorphisms with the risk of CRC in an Iranian population.

## MATERIALS AND METHODS

**Subjects: **The present population-based case-control study included a total of 204 (126 males, 78 females) patients with CRC and 239 (168 males, 71 females) disease free controls. All CRC patients were recruited from the chemotherapy department of Namazi hospital in Shiraz (Fars province, southern Iran). The mean age (SD) of the patients and the controls were 54.4 (14.0) and 53.4 (10.8) years, respectively. There was no significant difference in mean age between case and control groups (t=0.84, df=441, P=0.400). Iranian population is one of the most heterogeneous populations [11-13]. Therefore, we selected our patients and controls from the same ethnical religious group (Persian Muslims live in Fars province, southern Iran). Data on all CRC patients were obtained from personal interviews with patients. Informed consent was obtained from all Participants and the study was approved by the institutional review board at our University.

**DNA extraction and genotyping analysis: **Blood samples were obtained from the patient and control groups. Immediately after collection, whole blood was stored at −20°C until use. Genomic DNA for PCR was isolated from whole blood using the thawed blood samples by standard procedure [14]. The primers and PCR conditions for determining *SOD1 *A251G and *CAT *C-262T genotypes were the same as those reported previously [15-17] and shown in Table 1. For RFLP, the PCR products of *SOD1 *A251G and *CAT *C-262T SNPs were digested with *Msp*I (5U at 37°C for 16h) and *Eco*RV (5U at 37°C for 16h) (Fermentas), respectively.

**Table 1:** Primers used for *SODIA25aG* and *CAT C-262T* genotyping

**Genes**	**Sequences**	**Amplicon (bp)**	**T** **m ** **(°C)**
***SOD1***	F 5′-AGTACTGTCAACCACTAGCA-3′ R 5′-CCAGTGTGCGGCCAATGATG-3′	570	63
***CAT***	F 5-′CTGATAACCGGGAGCCCCGCCCTGGGTTCGGATAT- 3′ R 5′-CTAGGCAGGCCAAGATTGGAAGCCCAATGG-3′	190	68

**Statistical analysis: **Statistical analyses were performed with SPSS for Windows (version 17.0; SPSS Inc., Chicago, IL). A Chi-square test was performed for *SOD1 *and *CAT *polymorphisms to determine if the sample groups demonstrated Hardy–Weinberg equilibrium. The odds ratio (OR) and 95% confidence intervals (CIs) were calculated to estimate the association between the polymorphisms and CRC risk. For both polymorphisms, the more common allele was considered as the reference, whereas the less common allele was examined as the variant. Mean ± SD are presented for continuous variables. P-values of <0.05 were considered statistically significant.

## RESULTS

The genotype frequencies of the *SOD1 *A251G and *CAT *C-262T polymorphisms in the CRC and control groups are shown in Table 2. The genotypic frequencies for the both polymorphisms in cases (For *SOD1 *A251G polymorphism: χ2=1.78, df=1, P>0.05; For *CAT *C-262T polymorphism: χ2=1.08, df=1, P>0.05) and controls (For *SOD1 *A251G polymorphism χ2=0.11, df=1, P>0.05; For *CAT *C-262T polymorphism χ2=1.07, df=1, P>0.05) did not show significant deviation from expected frequencies based on Hardy-Weinberg equilibrium.

For *SOD1 *A251G polymorphism, the statistical analysis revealed that the AG *vs *AA genotype increased the risk of CRC (OR=1.85, 95% CI=1.14-3.02, P=0.013). Notably, the G allele (versus the A allele) significantly increased the risk of CRC (OR=1.71, 95% CI=1.09-2.69, P=0.019). However, there was no significant association between the* CAT *C-262T polymorphism and susceptibility to CRC, (T allele *vs *C allele, OR=0.86, 95% CI=0.62-1.20, P=0.403; CT+TT *vs *CC, OR=0.85, 95% CI=0.58-1.26, P=0.433) (Table 2).

**Table 2:** Distributions of the genotypes of the *SOD1 *A251G and *CAT *C- 262T polymorphisms in colorectal cancer (CRC) and control subjects

**Genotype**	**CRC (%)**	**Controls (%)**	**OR (95% CI)**	**P-value**
***SOD1*** ** A251G polymorphism**				
AA	155 (76.0)	204 (85.3)	1.0	-
AG	48 (23.5)	34 (14.3)	1.85 (1.14-3.02)	0.013
GG	1 (0.5)	1 (0.4)	1.31 (0.08-21.20)	0.846
AG+GG	49 (24)	35 (14.7)	1.84 (1.13-2.98)	0.013

***CAT*** ** C-262 C polymorphism**				
CC	132 (64.7)	146 (61.1)	1.0	-
CT	67 (32.)	85 (35.5)	0.87 (0.58-1.29)	0.499
TT	5 (2.5)	8 (3.4)	0.69 (0.22-2.16)	0.526

We also examined the combination effect of both *SOD1 *and catalase genetic polymorphisms on the CRC risk (Table 3). The results showed AG+GG genotype of *SOD1 *with CC genotype of catalase increases the rate of developing CRC (OR=2.38, 95% CI=1.25-4.52, P=0.008).

**Table 3:** Additive effect of the *SOD*1 and *CAT *genetic polymorphisms on the risk of colorectal cancer (CRC)

***SOD1***	***CAT***	**CRC**	**Controls**	**OR (95%)**	**P-value**
**AA**	**CT+TT**	59 (28.9)	78 (32.6)	1.0	-
**AA**	**CC**	96 (47.1)	126 (52.7)	1.01 (0.66-1.54)	0.974
**AG+GG**	**CT+TT**	13 (6.4)	15 (6.3)	1.14 (0.50-2.59)	0.744
**AG+GG**	**CC**	36 (17.6)	20 (8.4)	2.38 (1.25-4.52)	0.008

## DISCUSSION

CRC is one of the main causes of cancer mortality worldwide. The incidence of CRC is increased in Iran in recent years and CRC is the third and the fifth most common cancer in women and men respectively in Iran [1, 18]. The incidences of polymorphism in genomic DNA, and the risk of tumor progression in patients with cancer can vary substantially between different ethnic groups [19-21]. Although many polymorphisms are functionally neutral, some affect the level of gene expression or the function of the coded protein [22]. In our present study, we analyzed the association between *SOD1 *A251G and *CAT *C-262T polymorphisms with the risk of CRC in the Iranian population. We found that the *SOD1 *G allele was associated with an increased risk of cancer, and we found no statistically significant difference in *CAT *C-262T polymorphism between the controls and patients. These observations support the suggestion that genetic polymorphisms in antioxidant enzymes may alter the risk of cancer among persons with increased oxidative stress or decreased antioxidant capacity. Several different types of diseases, including diabetes, age-related diseases and cancers are dependent to defects in antioxidant pathways [8,23, 24]. Antioxidant enzymes can catalytically eliminate free radicals and other reactive species. Among the antioxidant enzymes, *SOD *is considered to be important in the oxidant defense mechanism, as it is involved in the first line of defense [25].

Many studies examining the association between ROS and various diseases have revealed that decreased antioxidant activity or excessive oxidative stress can cause several pathologic states [8, 23-29]. We also examined the combination effect of both *SOD1 *and *CAT *genetic polymorphisms on CRC risk and found that AG+GG genotype of *SOD1 *with CC genotype of *CAT *increases rate of developing CRC (Table 3). Therefore, the CC genotype compared to other genotypes of the *CAT *gene may increase the risk of CRC. In conclusion, our data provides evidence that the *SOD1 G *allele is associated with an increased risk of CRC in Iranian population. The Large population- based prospective studies with ethnically diverse populations are warranted to verify these findings.
